# Critical Appraisal of Evidence on Platelet-Rich Plasma and Stem Cell Therapy for Stress Urinary Incontinence: A Narrative Review

**DOI:** 10.7759/cureus.84009

**Published:** 2025-05-13

**Authors:** Muhammed Ishfaq, Praveen Gopi, Ahmad Omar, Michael S Floyd, Ekene Victor Ezenwa, Srinath Ravichandran, Kaylie E Hughes

**Affiliations:** 1 Department of Endourology and Reconstructive Urology, Mersey and West Lancashire Teaching Hospitals NHS Trust, Liverpool, GBR

**Keywords:** platelet-rich plasma (prp), regenerative medicine, rhabdosphincter, stem cell therapy, stress urinary incontinence

## Abstract

Stress urinary incontinence (SUI) is a common condition, affecting 20% of individuals. It is defined by the involuntary loss of urine during activities that increase intra-abdominal pressure, such as coughing or physical exertion. SUI may result from urethral hypermobility or intrinsic sphincter deficiency. Conventional treatments, including pelvic floor exercises, sling surgeries, and bulking agents, have varying success rates and complications. Emerging regenerative therapies such as platelet-rich plasma (PRP) and stem cell therapy (SCT) hold promise for improving urethral support and rhabdosphincter function.

This review critically examines recent evidence on the efficacy and safety of PRP and SCT for treating SUI. A thorough search of databases was conducted, focusing on clinical studies published on adult SUI patients treated with PRP and SCT interventions. Nine clinical trials met the inclusion criteria. All studies were conducted between 2011 and 2024. The keywords used were "Stress Urinary Incontinence", "Regenerative Medicine”, Cell- and Tissue-Based Therapy”, “Stem Cell Transplantation”, and “Stem Cell".

These studies investigated the use of PRP and mesenchymal stem cells, but inconsistencies in treatment protocols raise concerns about the reliability and generalizability of their findings. Additionally, there was a lack of detailed assessment tools to evaluate sphincter regeneration post-treatment and no standardized methods for PRP preparation.

This review describes the current status of stem cell research on SUI and suggests future directions to facilitate clinical applications. Future trials should follow a standardized protocol that includes detailed methods for stem cell or PRP preparation, dosage determination, application techniques, and necessary pre-procedural assessments. Objective outcome measures must incorporate improvements in urodynamic parameters, MRI evidence of sphincter regeneration, and comprehensive long-term follow-up evaluations. This approach ensures consistency and reliability in assessing treatment efficacy.

## Introduction and background

Urinary incontinence is a significant social concern affecting approximately 20% of women at some point in their lives, with a women-to-men ratio of 3:1 [1]. The International Continence Society defines urinary incontinence as the involuntary loss of urine, which can be a social or hygiene problem. The primary types of urinary incontinence include stress urinary incontinence (SUI), urgency urinary incontinence, mixed urinary incontinence, and continuous urinary incontinence.

SUI is specifically characterized by the involuntary loss of urine during physical exertion or increased intra-abdominal pressure, such as during coughing or sneezing [2]. Physiologically, SUI results from either urethral hypermobility, due to insufficient support for the urethra [3], or intrinsic sphincter deficiency [4]. These conditions may co-exist, but intrinsic sphincter deficiency is often considered more prominent than urethral hypermobility [5].

Pathophysiology of SUI

In cases of urethral hypermobility, the urethra becomes hypermobile due to a lack of supportive tissue. When intra-abdominal pressure increases, the urethra moves downward but its lumen does not close, leading to a lack of coaptation and urinary leakage [6].

Petros and Ulmsten's integral theory attributes this to the weakness of the bulbourethral ligament and the anterior vaginal wall [3], while DeLancey's hammock theory highlights insufficient support from the endocervical fascia and anterior vaginal wall [7]. McGuire introduced the concept of intrinsic sphincter deficiency, noting that some patients experience urinary incontinence despite a stable urethra during urodynamic fluoroscopic examinations [8]. He further developed the concept of abdominal leak point pressure (ALPP), which is the lowest abdominal pressure needed to overcome urethral resistance and cause urinary leakage in the absence of a bladder detrusor contraction [9].

SUI is often not caused by intrinsic sphincter deficiency alone but rather by a combination of urethral hypermobility and sphincter dysfunction. Daneshgari proposed a "trampoline theory" that suggests that SUI results from multiple contributing factors [10]. A diagnosis of SUI involves a thorough clinical history, bladder diary, physical examination, and dedicated investigations such as (a) Valsalva/cough test, (b) cotton tip test to assess urethral hypermobility, and (c) urinary PAD test to measure the amount of urine leakage. A bladder scan to assess post-void residual and urine culture are also mandatory investigations. Video-urodynamics (VUDS) remains the gold standard for visualizing urethral hypermobility and bladder neck closure, and measuring intravesical pressure (Pves), which is a sum of detrusor and abdominal pressures (Pabd). Urodynamics also evaluates the maximum urethral pressure using urethral pressure profilometry, with ALPP being a critical measure to assess the risk of incontinence. Fluoroscopy assesses the bladder neck and urethra during filling and voiding and screens for reflux [11]. In male patients, SUI can result from damage to the external sphincter following benign or malignant prostate surgery [12].

Treatment options in SUI

Several treatment options are available for managing incontinence, including pelvic floor physiotherapy, colposuspension to elevate the paraurethral tissue, and sling procedures that reinforce the pubourethral ligament with synthetic or autologous materials. Bulking agents assist in urethral coaptation.

Non-invasive incontinence treatments include pads, catheters, and pelvic floor muscle training. Invasive treatments include bulking agents, sling procedures, artificial urinary sphincters, and diversions [13], each of which is associated with variable outcomes.

Complications such as urinary retention, lower urinary tract symptoms, and genitourethral injuries can arise from these procedures. Issues such as non-sustainability of results and migration of bulking agents may necessitate repeat procedures or alternative treatments. Slings, once widely used, are now more restricted due to legal concerns over urethral injury. One of the key challenges in SUI surgery is balancing urethral resistance and bladder pressure while minimizing surgical morbidity [14].

Regenerative medicine in SUI

Regenerative medicine can theoretically restore the incompetent or lost intrinsic rhabdosphincter by injecting stem cell injection or platelet-rich plasma (PRP) into the paraurethral tissue. Stem cells have the ability to self-renew and differentiate [15]. PRP contains platelet concentration above normal and plasma-derived fibrinogen. Platelet-related growth factors that can regenerate damaged tissue have been widely used in the medical field [16]. However, bench-to-bedside challenges include harvesting appropriate stem cells that have the potential to transform into rhabdosphincteric cells without causing any other complications and with comparable results to existing therapies.

Adult stem cells are considered promising for regenerative therapy due to the advantages described below:

Sources: adult stem cells can be sourced from adipose tissue (fat), bone marrow, blood, and muscle. These sources make them accessible and relatively easy to harvest as opposed to embryonic stem cells.

Reduced cancer risk: adult stem cells have a lower risk of forming tumors compared to pluripotent stem cells (like embryonic stem cells). This is particularly important in regenerative medicine, where the potential for uncontrolled growth or malignancy must be minimized.

Autologous transfer: since adult stem cells can be harvested from the patient's own body (autologous transplantation), there is little to no risk of immune rejection. This makes adult stem cell therapy (SCT) safer, as there is no need for immunosuppressive drugs, which are often required in allogeneic (donor-based) transplants.

Ethical considerations: embryonic stem cells, which involve the destruction of embryos, have sparked significant ethical debates. Adult stem cells do not face the same ethical concerns. Their use typically avoids the moral dilemmas associated with using embryonic tissue.

Skeletal Muscle-Derived Cells for SUI

Skeletal and smooth muscles have been recognized as an essential source of progenitor or satellite cells responsible for muscle regeneration. MDSCs are a diverse group of multipotent cells with varying phenotypes based on their differentiation stage. They serve as precursors to various connective tissue cells, including myocytes and satellite cells. These cells can also give rise to mesenchymal, neuronal, and endothelial lineages, which are crucial for sphincter regeneration and the neural functions associated with continence [17,18]. These cells have the capacity to induce both structural and functional regeneration, as evidenced by significant improvements in the intravesical closure pressure of the urethra and a greater contractile function of the urethral sphincter [19].

Mesenchymal Stem Cells for SUI

Mesenchymal stem cells (MSCs) can be obtained from bone marrow, adipose tissue, umbilical cord, endometrial tissue, and oral mucosa. Beyond their regenerative properties, MSCs also possess anti-inflammatory and immunomodulatory properties.

ADSCs (adipose-derived stem cells) are among the most frequently used stem cells for both autologous and allogenic transplants due to their abundance and ease of extraction. As multipotent stromal cells, ADSCs have the ability to differentiate into adipogenic, chondrogenic, myogenic, and osteogenic cells. After periurethral injection and the application of induction factors, they can differentiate into myogenic cells, adopting a smooth muscle phenotype in around eight weeks. ADSCs have low immune rejection potential and prolonged proliferation at low serum conditions. ADSC is a promising treatment by enhancing elastin and smooth muscle content in urethral tissue, suggesting an improvement in urethral sphincter function.

MSCs such as bone marrow stem cells, umbilical cord blood stem cells, urine-derived stem cells, and amniotic fluid stem cells have been assessed in vitro and in vivo to treat urinary incontinence [20].

Multipotent endothelial progenitor cells can be isolated from peripheral blood to generate total nucleated cells, which can then be used in the treatment of urinary incontinence. The impact of growth factors and PRP has been explored in both in vitro and in vivo studies. This effect is linked to various growth factors secreted by platelets, including platelet-derived growth factor, transforming growth factor β1, fibroblast growth factors, and vascular endothelial growth factors [21]. PRP use has been researched in trauma patients and experimental trauma models [22]. Studies have demonstrated that the local application of autologous bone marrow-derived progenitor cells combined with PRP enhances tissue regeneration [23]. In the treatment of urinary incontinence, the regenerative properties of PRP help rebuild damaged tissues by supporting the urethra and urethral sphincter complex [24]. Given the potential regenerative effects of PRP, it is logical to inject PRP into the urethral sphincter to enhance sphincter muscle mass and increase urethral resistance.

The aim of this review is to critically appraise the available evidence in SCT and PRP for SUI.

## Review

Materials and methods

A comprehensive literature search was conducted using the Medline database with the following keywords: "Stress Urinary Incontinence", "Regenerative Medicine”, Cell- and Tissue-Based Therapy”, “Stem Cell Transplantation”. The eligibility criteria for inclusion were as follows: (1) adult patients (both male and female) aged 18 years or older with stress urinary incontinence, (2) the interventions of interest were SCT and PRP, with outcome measures focused on the efficacy and safety of these treatments, (3) studies published in the past 15 years, and (4) the use of PRP in conjunction with SUI treatment was an essential factor for inclusion in the review.

Studies were excluded if they were preclinical, editorials, research letters, non-English, animal studies, or unpublished works with only abstracts available.

The literature search was first performed on PubMed and Cochrane database using the keywords "Stress Urinary Incontinence", "Regenerative Medicine”, Cell- and Tissue-Based Therapy”, “Stem Cell Transplantation”, and “Stem Cell". In the initial search, eight articles were identified. Additionally, a further search was conducted on Google Scholar using the keywords "platelet-rich plasma” and “urinary incontinence", which led to the inclusion of one more relevant study published in a high-impact journal. These studies (Tables 1-4) were subsequently analyzed and included in the final review.

**Table 1:** Study characteristics

Reference	Ethical approval and informed consent	Country	Study period	Study design	Journal
Long et al., 2021 [25]	Yes	Taiwan	2018	Prospective intervention study	Nature
Lee et al., 2021 [26]	Yes	Taiwan	2018-2020	Prospective interventional study	Nature
Garcia-Arranz et al., 2020 [27]	Yes	Spain	2011-2014	Prospective, non-randomized phase I‐IIa clinical trials	Stem Cells Translational Medicine
Sharifiaghdas et al., 2019 [28]	Yes	Iran	2013-2016	Prospective single-arm clinical trial	Urology Journal
Gotoh et al., 2020 [29]	Yes	Japan	2017-2019	Prospective, multicenter, single-arm study	International Journal of Urology
				Clinical trial	
Pourmand et al., 2017 [30]	Yes	Iran	August to December 2012	Prospective intervention studies	Acta Medica Iranica
Chiang and Kuo, 2022 [31]	Yes	Taiwan	April 2018 to October 2020	Prospective intervention study	Frontiers in pharmacology
Daneshpajooh et al., 2022 [32]	Yes	Iran	2017-2019	Prospective randomized clinical trial	Journal of Stem Cells & Regenerative Medicine
Schmid et al., 2023 [33]	Yes	Switzerland	January 2020 to September 2021	Prospective randomized clinical trial – phase I	International Urogynecology Journal

**Table 2:** Population characteristics *Not included in the study RP, radical prostatectomy; Holep, holmium enucleation of the prostate; UI, urine incontinence; ISD, intrinsic sphincter deficiency

Reference	n	Sex	Age	BMI (kg/m^2^)/weight (kg)	Risk factors/medical history
Long et al., 2021 [25]	20	Female	44.5 ± 9.1	BMI 22.7 ± 6.3	Parity
Lee et al., 2021 [26]	28	Male	71.8± 8.9	*	Postprostatectomy
Garcia-Arranz et al., 2020 [27]	19	Male (n=9), Female (n=10)	Male (67.6 ± 5), female (56.8 ± 9)	*	*
Sharifiaghdas et al., 2019 [28]	20	Female	51.5 (30–70)	Mean weight: 74.2	race, parity
Gotoh et al., 2020 [29]	45	Male	70.3	*	RP/HoLEP
Pourmand et al., 2017 [30]	10	Female	45.8	Mean weight: 71.8	History of UI, pelvic organ prolapse, hysterectomy
Chiang and Kuo, 2022 [31]	26	Female	61.7 ± 15.3 (20–88)	*	ISD, previous anticontinence surgery
Daneshpajooh et al., 2022 [32]	30	Female	48.9 ± 2.76 (stem cell group), 45.7 ± 1.99 (sling surgery group)	*	Parity
Schmid et al., 2023 [33]	10	Female	45 (32–58)	Median BMI 24	*

**Table 3:** Intervention characteristics ADRC, autologous adipose-derived regenerative cell; ASC, adipose-derived stem cell; MDSC, mesenchymal-derived stem cell; MPC, muscle precursor cell; PRP, platelet-rich plasma

Reference	Cell type	Cell origin	Trans or periurethral	Number of treatments	Anesthesia	Additional treatments
Long et al., 2021 [25]	PRP	Blood	Periurethral (mid urethra)/transvaginal	Single dose, 5 mL at 3 points, monthly for 3 months	No anesthesia	No additional treatment
Lee et al., 2021 [26]	PRP	Blood	Urethral sphincter injections Under cystoscopically	4 doses (5 points), 4 treatments/month for 3 months	General anesthesia	No additional treatments
Garcia-Arranz et al., 2020 [27]	ASCs	Liposuction from the subcutaneous tissue	Intraurethral, cystoscopically, 7-8 injections from the bladder neck to the mid-urethra.	Male, single dose 20 x10^6^ ASCs and second dose of 40 x10^6^ at 3 months if no improvement achieved. Female, single dose, single treatment 40 x10^6^ ASCs	Under sedation	No additional treatments
Sharifiaghdas et al., 2019 [28]	MDSCs	Quadriceps femoris muscles	Transurethral 2 positions	Single dose, single treatment >50,000000	Local anesthesia	No additional treatment
Gotoh et al., 2020 [29]	ADRCs and adipose tissue	Liposuction from abdominal wall	Periurethral, region of the external urethral sphincter	Single treatment, cystoscopically at 4,5 and 7,8 position 20 mL	General anesthesia	No additional treatment
Pourmand et al., 2017 [30]	ASCs	Abdominal subcutaneous adipose tissue	Trans and periurethral 2 injections	Single dose treatment 1,180,000 cells/mL, 10 mL	Local anesthesia	No additional treatment
Chiang and Kuo, 2022 [31]	PRP	Blood	Periurethral, 5 sites, monthly treatment for 3 months	5 mL at 5 points, total 4 treatments within 3 months	General anesthesia	No additional treatment
Daneshpajooh et al., 2022 [32]	MDSC	Muscle and fibroblast	Periurethral, 4 sites, mid urethra at sphincter level, cystoscopically	Single dose of 30 million MDCs and 30 million fibroblasts	Spinal anesthesia	Control group (n=15) underwent mid urethral sling surgery
Schmid et al., 2023 [33]	MPC	Muscle	Transurethral, under trans vaginal US guidance, intra sphincteric injections	8-100 x 10^6 ^MPCs, single injection	General anesthesia	Control group (n=5) received neuromuscular electromagnetic stimulation

**Table 4:** Outcome characteristics ALPP, abdominal leak point pressure; FSFI, Female Sexual Function Index; FUL, functional urethral length; GRA, Global Response Assessment; ICIQ-SF, International Consultation on Incontinence Questionnaire Short Form; ICIQ-UI-SF, International Consultation on Incontinence Questionnaire–Urinary Incontinence Short Form; IIQ-7, Incontinence Impact Questionnaire; MCUP, maximum urethral closure pressure; OABSS, Overactive Bladder Symptom Score; POPDI-6, Pelvic Organ Prolapse Distress Inventory-6; PVR, post-void residual; SF-36, Short Form Health Survey Questionnaire; UDI-6, Urinary Distress Inventory; UDS, urodynamics; UI, urinary incontinence; VAS, visual analog scale; VDUS, video-urodynamics

Reference	Follow-up duration	Outcome tests	Efficacy outcomes
Long et al., 2021 [25]	6 months	ICIQ-SF, UDI-6, IIQ-7, OABSS, POPID-6, FSFI	n=20: 12 (improved) 8 (unchanged or worse)
Lee et al., 2021 [26]	13 .4 ± 5.7 months	GRA, VAS, VUDS, ALPP	n=28: n=6, complete continence (21.4%); n=26, clinical improvement GRA ≥ 1; n=2, no improvement
Garcia-Arranz et al., 2020 [27]	12 months	SF-36, ICIQ-SF, clinical history, clinical examination, biochemical and hematological features, urine culture, cystoscopy and UDS	Male (n=10): 2 excluded (one alternative diagnoses, one contaminated liposuction), 8 treated. 2/8 no UI, 6/8 UI. Females (n=10): 5/10 no UI, 5/10 UI
Sharifiaghdas et al., 2019 [28]	24 months	Validated questionnaires (IIQ-7, UDI-6)	n = 20: withdrawal in 3 patients, complete response in 5 patients, treatment failure in 12 patients
Gotoh et al., 2020 [29]	52 weeks	A 24-h urine pad test	n=43 received ADRCs, 37.2% (95% CI 23.0–53.3%) showed improvement
Pourmand et al., 2017 [30]	24 weeks	Validated questionnaires (ICIQ)	An overall decrease of UI in patients
	n = 10	24-h pad test	Decrease of approximately 20 g in pad tests
Chiang and Kuo, 2022 [31]	12 months	GRA, UDI-6, IIQ-7, VAS of SUI, VUDS	12 (46.2%) were totally dry at 3 months
Daneshpajooh, Azar et al., 2022 [32]	12 months	ICIQ-UISF, I-QOL, clinical examination, cough Test, 1-h pad test	Stem cell group (n=15): 10 (66.6%) showed improvement at 1 year and 33.3% had full recovery. Mid-urethral sling group (n=15): 13 (93.3%) showed improvement at 1 year and 80% had full recovery.
Schmid et al., 2023 [33]	6 months	PVR, MUCP, FUL, maximum bladder capacity, pad usage, ICIQ-UI-SF, MRI of the pelvis	Improved QoL, 2/3 patients were dry, increase in EUS on MRI

Discussion

Regenerative medicine represents a promising frontier in the management of SUI, particularly as an alternative to traditional surgical and pharmacologic treatments.

Long et al. [25] found that 12 (60%) out of 20 patients experienced significant improvement in their SUI following treatment (Table 4). The study presents several notable strengths, particularly in its innovative use of autologous platelet-rich plasma (A-PRP), a novel and minimally invasive treatment approach for SUI. This is the first study to report on the clinical outcomes of A-PRP for SUI in women, highlighting its potential as a non-surgical alternative. The treatment was shown to be safe, with no adverse reactions, and led to significant symptom improvement at both one and six months, as assessed using validated questionnaires. Additionally, the autologous nature of PRP minimizes the risk of immune reactions, making it a favorable option compared to synthetic bulking agents. The study also observed a potential trend indicating that younger patients (under 40 years) responded more favorably to treatment, offering insight into future patient selection criteria. Moreover, the inclusion of a secondary analysis on sexual function adds further depth to the study’s findings.

However, the study by Long et al. [25] has several limitations that affect the reliability and generalizability of its conclusions. The small sample size of only 20 patients restricts statistical power and limits the ability to draw definitive conclusions. The absence of a control group hinders comparisons with other treatment modalities and does not account for placebo effects. Furthermore, the short follow-up period of six months precludes assessment of long-term efficacy. Limited urodynamic data (available from only eight patients) reduces the capacity to evaluate the physiological effects of A-PRP. Additionally, the study did not provide details regarding pre-treatment symptom duration or prior interventions, which are important factors in outcome interpretation.

The study [25] also failed to control for potential confounding variables, including parity, menopausal status, and underlying medical conditions, all of which could influence treatment outcomes. The use of self-reported questionnaires as subjective outcome measures introduces the potential for bias. Moreover, the lack of a standardized PRP preparation protocol raises concerns regarding reproducibility. Cultural factors and the specific patient population in Taiwan may further limit the generalizability of the findings. The International Consultation on Incontinence Questionnaire-Urinary Incontinence Short Form (ICIQ-SF), used to assess incontinence severity, does not account for fluid intake or patient activity levels - variables that could influence outcomes. The Stamey classification, which evaluates severity based on triggering activities, may have been a more appropriate tool. Additionally, the study did not provide radiological or biological evidence of urethral sphincter regeneration - one of the primary objectives. It is possible that observed improvements were due to a bulking effect rather than true tissue regeneration. Lastly, the cost and accessibility of A-PRP treatment were not addressed, which could affect its practical application. Despite these limitations, the study provides preliminary evidence supporting the use of A-PRP for the treatment of mild-to-moderate SUI. Further research with larger, randomized controlled trials and longer follow-up is necessary to confirm these findings.

Lee et al. [26] reported significant results of PRP injection in male patients who had undergone radical prostatectomy for prostate cancer (Table 2). The group reported 21.4% (n=6) achieving complete continence after multiple cystoscopic injections under general anesthesia. VDUS was used to confirm SUI after all conservative measures had failed. However, they did not mention the prostate cancer stage or the post-operative prostate-specific antigen levels. There was also no mention of pre-operative lower urinary tract symptoms (LUTS), which are important as histopathological nerve-sparing and wide margins may affect post-operative urinary continence outcome. The Global Response Assessment scale and visual analog scale were used for assessment, but no mention was made of whether the patients understood the questions or if the questionnaires were validated in the patients' language. The Stamey classification for incontinence was applied pre-operatively but not post-operatively. Additionally, the quality of the PRP intervention was not assessed with biological tests to determine the platelet and component content, nor pre- nor post-operative imaging or sphincter biopsy was performed to quantify the effect on urethral sphincter regeneration, which was the primary aim of the study. There was also no subgroup analysis for open, laparoscopic, or robotic radical prostatectomy. The data were analyzed using the Wilcoxon sum test, but a paired t-test would have been more appropriate based on the data.

Garcia-Arranz et al. [27] reported using adipose-derived stem cells (ASCs) for treating SUI in both males and females by injecting cells from the bladder neck to the mid-urethra (Table 3). They reported improvements in urinary incontinence in 37.5% (n=2) of the male group and 50% (n=5) of the female group (Table 4). The study assumed that sphincter damage was the cause of incontinence, but no imaging or sphincter biopsy was performed to confirm this, raising questions about the study's rationale. The male participants had undergone radical prostatectomy, but the procedural specifics (open, laparoscopic, or robotic) were not mentioned. Additionally, no pre-intervention data, including surgical margins status, were provided. There were also no data about pre-operative LUTS or incontinence, which could affect the outcome and contribute to treatment failure. SUI requires VUDS, but the study only performed standard urodynamics. There was no mention of ALPP, a critical parameter for SUI diagnosis. No pre- and post-surgical comparisons of VUDS outcomes were available. In the female trial, there was no description of female SUI or whether concomitant urgency or other LUTS had been excluded. The lack of VUDS for diagnosing SUI raises doubts about the accuracy of the diagnosis.

Sharifiaghdas et al. [28] reported using autologous muscle-derived stem cells (MDSCs) to treat SUI in females by injecting them into the urethra (Table 3). They found that 29% (n=5) of patients had a complete cure. However, no description of previous treatments was provided, and no VUDS was performed to confirm the diagnosis of SUI. While urodynamics was performed, there was no explanation as to why ALPP, an essential parameter for SUI diagnosis, was not measured, raising concerns about the accuracy of the SUI diagnosis. All questionnaires were in English, and no information was provided regarding whether patients understood the questions or whether the questionnaires were translated and validated. No pre- or post-intervention imaging or sphincter biopsy was performed to confirm the regenerative effect of MDSCs on the urethra. The study did not specify which statistical tests were used to analyze the data, and ALPP was not analyzed pre- and post-intervention.

Gotoh et al. [29] reported autologous adipose-derived regenerative cell (ADRC) injections for post-prostatectomy and holmium laser enucleation of prostate (HoLEP)-related SUI in males. However, these were two different groups, and data were not analyzed separately. No pre-operative clinical data, such as LUTS, were available for the HoLEP group No objective evidence of sphincter deficiency was provided, and the validity of the Celution® system used for ADRC isolation was not described. Improvements in daily urinary leakage were measured using the pad test, but this test is dependent on factors such as fluid intake and daily activities, which were not standardized in the study. A reduction of 57.7% (n=10) in pad test results over 52 weeks was reported, and the change is not necessarily clinically significant. The number of pads used daily, which is a more important measure than the amount in grams, was not reported. No imaging or histopathology was performed to confirm sphincter regeneration, which was the primary goal of the study. ALPP was only measured in 23 patients at the end of the study (Table 4).

Pourmand et al. [30] reported using ASCs to treat female SUI, with 10 patients followed for 24 weeks. Sphincter deficiency was assumed to be the cause of SUI, but no imaging or urodynamics tests (such as ALPP or VDUS) were performed to confirm sphincter deficiency. The regenerative ability of the cells was not adequately explained, and no in vivo or in vitro evidence of urethral sphincter regeneration was provided. Statistically, the study showed a decrease in urinary incontinence using the pad test and ICIQ-SF, but no details on the statistical analysis were provided.

Chiang and Kuo [31] explored the effectiveness and safety of repeated PRP urethral sphincter injections for treating SUI in women. The study's strengths include a relatively large sample size of 21 women and a follow-up period of up to 12 months, showing sustained improvements in symptom scores and urodynamic parameters, with 26.9% of women remaining continent after one year. Additionally, the study suggests that PRP may promote urethral sphincter regeneration, distinguishing it from other treatments such as urethral bulking agents. However, key limitations include the lack of a control group, reliance on subjective outcome measures, and an unclear mechanism of action for PRP's effects. The study also highlights potential confounding factors in non-responders and variability in response based on baseline characteristics. Further analysis of the data was limited, as improvements were not clearly defined, and a non-parametric test such as the Wilcoxon rank-sum test was used when a paired t-test would have been more appropriate for analyzing ordinal data such as the severity of incontinence. Other studies, including those by Garcia-Arranz et al.[27] and Sharifiaghdas et al. [28], faced comparable limitations: they did not utilize imaging or biopsies to verify sphincter regeneration, lacked VDUS for diagnosing SUI, and did not measure ALPP.

Daneshpajooh et al. [32] compared periurethral injection of autologous muscle-derived stem cells and fibroblasts with mid-urethral sling surgery in women with SUI and offered valuable insights into the potential of regenerative therapies. Among its strengths, the study employed rigorous inclusion and exclusion criteria, matched participants on key variables, and utilized both subjective (ICIQ-UISF, I-QOL) and objective (cough stress and pad tests) outcome measures, ensuring comprehensive assessment. The cell therapy approach is less invasive than surgery and aligns with the goal of tissue repair rather than merely symptom management, potentially reducing long-term complications associated with synthetic materials. However, the study has notable limitations. The sample size was relatively small (30 patients), which may limit the generalizability of the findings and the statistical power to detect differences. It was unclear whether the randomization process by random digit table was performed manually or using computerized methods. The follow-up period, while extending to 12 months, may still be insufficient to assess long-term efficacy and safety, particularly for regenerative treatments. Additionally, the control group was open-label, and biopsy was not performed on these patients, introducing potential bias. The results showed that while both groups improved, the standard mid-urethral sling group had significantly better outcomes at 6 and 12 months, raising questions about the comparative effectiveness of cell therapy in its current form. Further large-scale, blinded studies with longer follow-up are needed to validate these findings and optimize cell-based interventions for SUI.

The phase I clinical trial conducted by Schmid et al. [33] demonstrated that transurethral injection of autologous muscle precursor cells (MPCs) into the external urinary sphincter is a safe and feasible treatment for female SUI. The study's strengths include its prospective, randomized design, rigorous inclusion and exclusion criteria, and the use of both objective (urodynamic studies, MRI) and subjective (ICIQ-UI-SF questionnaire) outcome measures (Table 4), which provide a comprehensive assessment of safety and preliminary efficacy. Additionally, the minimally invasive nature of the procedure and the use of autologous cells reduce the risk of immunogenic reactions and severe adverse events, as reflected by the minimal and manageable complications observed. The long-term follow-up plan for five years is appreciable.

However, the aforementioned study [33] has several limitations. The small sample size (n=9 treated patients) limits the statistical power and generalizability of the findings, and the trial was not powered for secondary efficacy outcomes, necessitating cautious interpretation of these results. The short follow-up period (six months) may not capture long-term safety or durability of the therapeutic effect. Furthermore, the absence of subgroup analysis between the MPC and MPC+NMES (neuromuscular electromagnetic stimulation) groups and the lack of a control group restrict the ability to attribute observed improvements solely to the intervention. Larger, controlled studies with extended follow-up are needed to validate these promising preliminary results and assess long-term efficacy and safety.

Suggestions for future study design

To improve study designs, it is essential to accurately diagnose intrinsic sphincter deficiency as the cause of SUI. Studies should include the use of VDUS and measurement of ALPP, along with imaging such as MRI to assess sphincter regeneration before and after the intervention. Exclusion or standardization of pre-intervention LUTS and activity levels, along with the classification of SUI severity using the Stamey classification system in addition to pad tests, should be included. Outcome measurements should include a complete analysis of all data to demonstrate the intervention's effect, focusing on clinical benefits rather than statistical significance. A control group receiving standard therapy should be included for comparison, and ethical concerns regarding the lack of standard therapy should be addressed.

## Conclusions

In conclusion, while PRP or SCT appears to be novel treatment for SUI, more rigorous studies with defined entry criteria are required, including BMI and previous treatments. Future research should include a comprehensive understanding of SUI, objective diagnostic tools such as VUDS and imaging for sphincter assessment, and the measurement of key parameters such as ALPP. Well-defined outcome measures should be established pre-operatively, taking clinical benefits into account. Larger trials should include control groups and a standardized approach to PRP and stem cell treatments. Combining PRP and stem cells may be an effective avenue for future studies in comparison to existing therapies.
